# A Case of Femoral Artery Aneurysm Caused by Methotrexate Associated Lymphoproliferative Disease

**DOI:** 10.7759/cureus.57933

**Published:** 2024-04-09

**Authors:** Atsushi Harada, Masahiro Nishihara, Hiromichi Takahashi, Yojiro Machii, Masashi Tanaka

**Affiliations:** 1 Department of Cardiovascular Surgery, Nihon University School of Medicine, Tokyo, JPN; 2 Department of Rheumatology and Collagen Medicine, Nihon University School of Medicine, Tokyo, JPN; 3 Department of Hematology and Oncology, Nihon University School of Medicine, Tokyo, JPN

**Keywords:** oral methotrexate, : rheumatoid arthritis, deep vein thrombosis (dvt), femoral artery aneurysm, methotrexate-associated lymphoproliferative disease

## Abstract

An 82-year-old man with left leg edema was referred to our department after an ultrasound examination by his previous physician, which revealed deep vein thrombosis (DVT) in the left superficial femoral vein and a left common femoral artery aneurysm (CFAA). The DVT was caused by the CFAA. The patient was adjudged to be at high risk of peripheral embolization due to the irregular shape of the varicose vein and a large amount of mural thrombus.

Surgery was performed to replace the artificial blood vessel. The patient displayed firm adhesion to the surrounding area, marked lymph node swelling, and a large amount of mural thrombus in the mass. The superficial femoral artery (SFA) demonstrated severe intimal thickening and partial dissection.

The postoperative course was good, and the patient was undergoing rehabilitation to be discharged home; however, B-cell lymphoma was suspected based on the pathology results of the mass wall submitted intraoperatively.

The patient had a history of rheumatoid arthritis and was treated with methotrexate (MTX). During the course of his illness, a subcutaneous mass was found on his right forearm, and a skin biopsy revealed MTX-associated lymphoproliferative disease (MTX-LPD), which had resolved with MTX withdrawal. The histopathological results of the skin biopsy matched those of the CFAA mural thrombus, and Epstein-Barr virus-positive cells were also observed, leading to the diagnosis of MTX-LPD, which was considered to be the cause of CFAA. No MTX-LPD was identified in the vessel walls or intramural thrombus. We herein report a case of CFAA with an extremely rare etiology and clinical presentation.

## Introduction

Common femoral artery aneurysms (CFAAs) occur primarily in the common femoral artery (CFA) and rarely in the superficial femoral artery (SFA) or deep femoral artery (DFA) [1]. CFAAs may be recognized by the presence of a pulsating mass, peripheral embolization of a mural thrombus, rupture, or venous compression, but they are often asymptomatic [2]. They are classified as true aneurysms or pseudoaneurysms, with pseudoaneurysms being more common. Pseudoaneurysms are most frequently iatrogenic, such as after catheterization or endovascular treatment. The indications for treatment are symptomatic aneurysms or aneurysms >2.5 cm in diameter [3]. Endovascular treatment can be considered, but replacement with an artificial vessel is recommended. In this case report, we describe a sporadic case of CFAA surgery with methotrexate (MTX)-associated lymphoproliferative disease (MTX-LPD) as the probable cause.

## Case presentation

The patient was an 82-year-old man with a history of hypertension, diabetes mellitus, atrial fibrillation, cerebellar infarction, cataracts, and rheumatoid arthritis (RA). The patient was diagnosed with MTX-LPD. MTX was withdrawn, and the mass shrunk thereafter. He had visited his previous doctor with a chief complaint of left leg edema, and ultrasonography showed a thrombus in the left superficial femoral vein (SFV) and the left common femoral artery aneurysm(CFAA).

**Figure 1:**
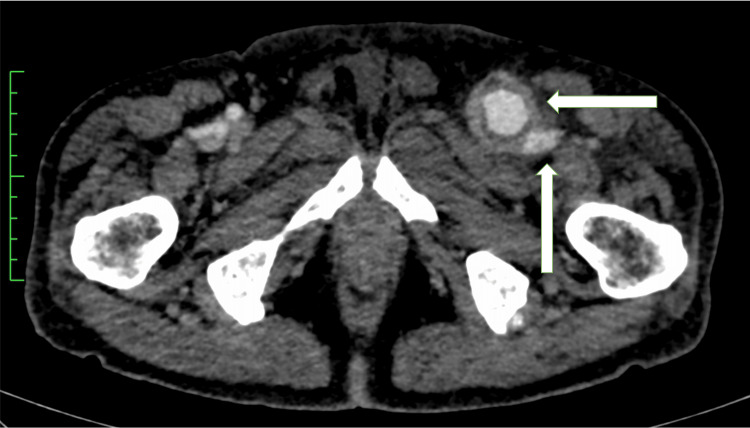
The preoperative CECT image On the preoperative CECT image, the left CFA shows an enlargement of 3.1×3.4 cm in diameter, marked wall thickening, and enlarged surrounding lymph nodes (⇦), while the left SFV is compressed (⇧). CECT - contrast-enhanced computed tomography; CFA - common femoral artery; SFV - superficial femoral vein

**Figure 2:**
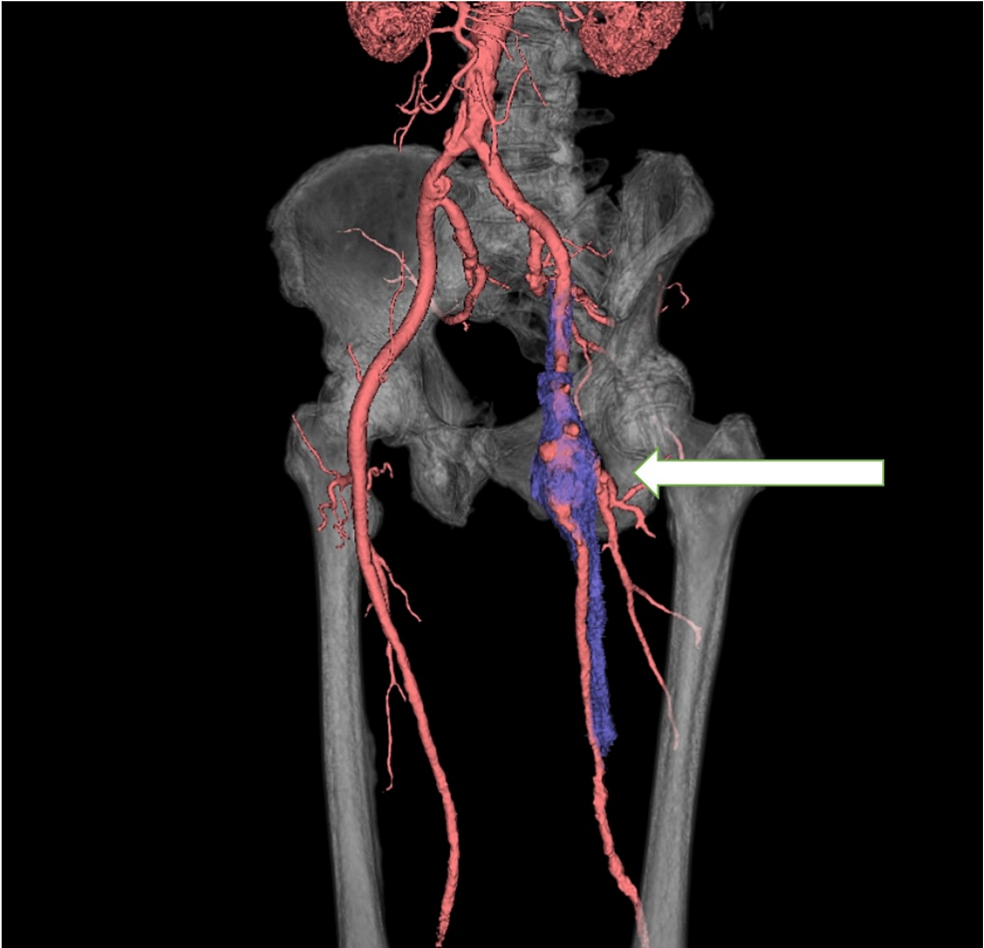
3D constructed image of preoperative CT Irregular widening from the left CFA to SFA and DFA (⇦). CT - computed tomography; CFA - common femoral artery; SFV - superficial femoral vein; SFA - superficial femoral artery; DFA - deep femoral artery

Contrast-enhanced computed tomography (CECT) showed that the left common femoral vein compression by the CFAA was the cause of the deep venous thrombosis (DVT), given the high risk of peripheral embolization of the mural thrombus. Moreover, because the aneurysm extended into the SFA and DFA, we decided on artificial vessel replacement rather than endovascular treatment due to the anatomy of the patient (Figures 1-2).

The patient had no history of CFA puncture, including catheterization or endovascular treatment, and CT showed severe wall thickening and marked surrounding lymph node enlargement. The findings revealed no aneurysms in the aorta, peripheral, or visceral arteries.

**Figure 3:**
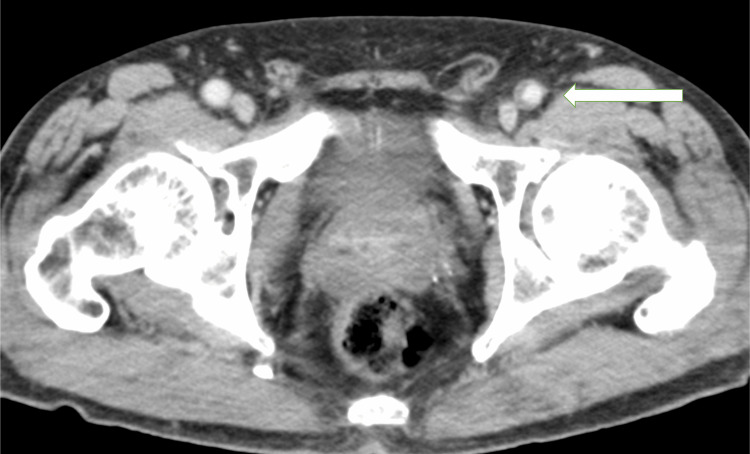
The previous CT image performed at our hospital Left CFA is not aneurysmal, but a mural thrombus is observed. A timer is thought to have developed in the mural thrombus and rapidly formed an aneurysm (⇦). CT - computed tomography; CFA - common femoral artery

CFA was normal in diameter with no mass formation. However, there was a thrombus in the wall on a previous CT scan taken at our hospital, and we suspected a recent rapid mass formation and a tendency toward enlargement (Figure 3).

Blood tests showed average white blood cell counts but elevated levels of C-reactive protein (CRP; 5.61 mg/dL), D-dimer (18.7 μg/mL), and lactate dehydrogenase (LDH; 324 U/L). Other blood tests showed no abnormalities, nor did the results of preoperative chest radiography, transthoracic echocardiography, pulmonary function testing, ankle brachial pressure index (ABI), carotid ultrasonography, and myocardial scintigraphy. Electrocardiography showed atrial fibrillation, which had been previously noted, and the patient was taking anticoagulant medication with 2.5 mg of apixaban twice daily.

**Figure 4:**
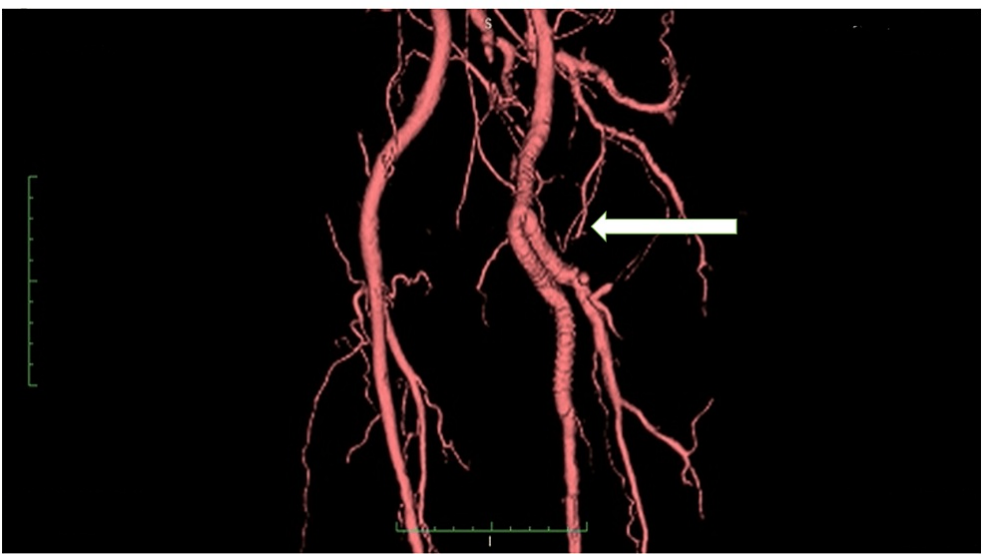
Postoperative CT No residual disease, no anastomotic stenosis (⇦). CT - computed tomography

The patient was operated on in the supine position under general anesthesia, with firm adhesions around the left CFAA and marked lymphadenopathy. A large amount of mural thrombus was found in the mass. The mass wall and mural thrombus were submitted to histopathology. The SFA was partially dissected with severe intimal thickening. The bypass from CFA to SFA was performed with end-to-end anastomosis using an 8-mm PROPATEN (Gore Medical, Flagstaff, AZ, USA) vascular graft. From the middle of the graft of the bypass from CFA to SFA, side-to-end anastomosis was performed using PROPATEN until DFA (Figure 4). We used polytetrafluoroethylene suture for sutures.

The postoperative course was good; the ABI was 1.17/1.16, with no reduction, while CT and ultrasonography showed no stenosis at the anastomosis of the artificial vessel, a normal flow velocity of the bypass graft, and good blood flow to the toe. The superficial femoral vein was decompressed, and DVT and leg edema tended to improve.

**Figure 5:**
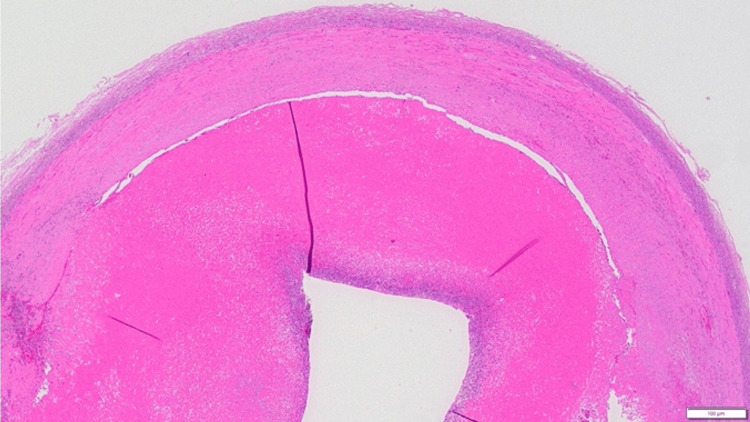
Pathology of the CFAA wall A highly thickened arterial wall intima is noted. Scattered foci of atypical cells are noted just below the endothelial cells and are accompanied by severe degenerative necrosis. CFAA - common femoral artery aneurysm

**Figure 6:**
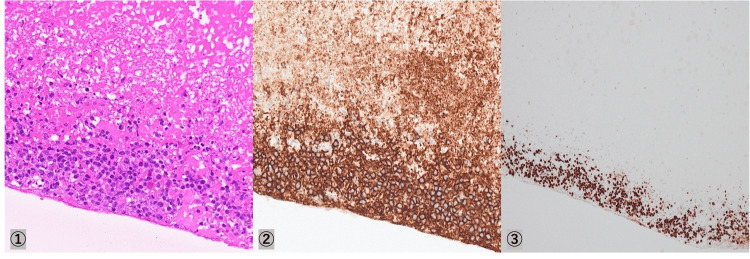
Pathology of the CFAA wall Pathological images of CFAA performed at our hospital (① HE (×20), ② CD20, ③ Ki67). CFAA - common femoral artery aneurysm; HE - hematoxylin and eosin; CD - cluster of differentiation; Ki67 - marker of proliferation Ki-67

Intraoperatively submitted mural thrombus and atypical lymphocytic infiltrate from the aneurysmal wall, positive B-lymphocyte markers (cluster of differentiation (CD)20, CD79a, CD21), negative T lymphocyte markers (CD3, CD5, CD10), and Epstein-Barr (EB) virus-positive cells (Figures 5-6). 

**Figure 7:**
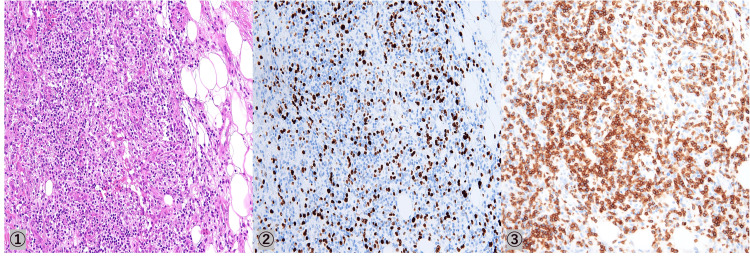
Pathological image of skin biopsy performed by the previous hospital ① HE (×20), ② CD20, ③ Ki67 HE - hematoxylin and eosin; CD - cluster of differentiation; Ki67 - marker of proliferation Ki-67

The pathology was consistent with a skin biopsy performed by a previous physician, and a diagnosis of MTX-LPD was made (Figure 7). 

Positron emission tomography-CT was performed, but no accumulation was observed, and the disease was considered to be under control; therefore, we decided to follow up with MTX withdrawal.

After discharge from the hospital, the patient was regularly monitored in our outpatient clinic. As of now, 10 months have passed, with no stenosis of the bypass graft or anastomotic site and no recurrence of aneurysm.

## Discussion

The presence of MTX-LPD from the mid-artery aneurysms is considered to be very rare and has not been reported previously.

MTX-LPD is a lymphoma that appears with long-term administration of MTX, an antirheumatic drug, with no known cause. It should always be suspected in patients with RA who present with unexplained fever, enlarged lymph nodes, hepatosplenomegaly, elevated LDH, or elevated CRP during MTX administration.

Spontaneous regression of lymph nodes is observed in approximately 30% of cases within weeks to months after MTX discontinuation, although approximately half of these cases relapse [4].

Diffuse large B-cell lymphoma (DLBCL) accounts for 35-60% of MTX-LPD cases, followed by classical Hodgkin's lymphoma in 12-25%, while other subtypes of lymphoma are rare. Their histopathologic features are similar to those of immunocompromised patients with comparable lymphoma subtypes [5]. Pleomorphic or lymphoplasmacytic infiltrates have also been reported in 20% of MTX-LPD cases, and EB virus is detected in 25-60% of MTX-DLBCL pleomorphic infiltrates [6].

CFAAs are often diagnosed by pulsatile mass, rupture, or leg ischemia and are classified as accurate or pseudoaneurysms, with pseudoaneurysms being more common. Pseudoaneurysms are most frequently iatrogenic, such as after catheterization or endovascular treatment. Piffaretti et al. reported that 57% of aneurysms were in the CFA, 26% were in the SFA, 17% were in the DFA, 26% were bilateral, and 48% were aneurysms in other sites. As for aneurysms at other sites, the abdominal aorta is reported to be the most common (60%), followed by the popliteal artery (31%) [1].

In this case, the CFAA was a true solitary aneurysm in the left CFA, with no other aneurysms.

The patient had a right forearm mass caused by MTX-LPD and was treated with MTX withdrawal. In this case, atypical lymphocytes were found in the mural thrombus within the CFAA, and the pathology showed infiltration of the aneurysm wall. The pathology was consistent with that of the skin biopsy diagnosed as MTX-LPD, which was positive for B-lymphocyte markers (CD20, CD79a, CD21), negative for T-lymphocyte markers (CD3, CD5, CD10), and positive for EB virus. The clinical and pathological findings of this case provide a reason to support MTX-LPD infiltration of the aneurysm.

Intravascular large B-cell lymphoma (IVLBCL) is a disease in which tumor lymphocytes are found in blood vessels. IVLBCL can cause embolization in small peripheral arteries, resulting in ischemic lesions and pulmonary embolism [7]; however, there have been no reports of an aneurysm or aneurysmal lesions. The involvement of the CFA, a medium-sized artery with a high blood flow rate, as in this case, has not been reported and is considered to be extremely rare.

CT previously taken at our hospital showed left CFA with standard diameter but wall thrombus.

In the present case, MTX-LPD infiltrated into the mural thrombus and caused rapid aneurysmal growth. It is possible that MTX-LPD also infiltrated the central and peripheral vessels that were replaced by artificial vessels in this case, and careful follow-up is required in the future.

## Conclusions

We experienced a case of CFAA, possibly due to the infiltration of MTX-LPD into a mural thrombus. This is a scarce condition, and preoperative diagnosis was difficult. Fortunately, after artificial vessel replacement and MTX withdrawal, the patient is doing well with no symptom recurrence, but there is still a possibility of symptom recurrence or the appearance of new vascular lesions, and careful follow-up is necessary. If vascular invasion is observed again, chemotherapy should be considered in addition to revascularization. Since perioperative management may differ depending on the cause of the mass, an accurate diagnosis with the patient's history in mind is essential for an excellent surgical outcome.
